# Full controlling of Fano resonances in metal-slit superlattice

**DOI:** 10.1038/srep18461

**Published:** 2015-12-18

**Authors:** Zi-Lan Deng, Natesan Yogesh, Xiao-Dong Chen, Wen-Jie Chen, Jian-Wen Dong, Zhengbiao Ouyang, Guo Ping Wang

**Affiliations:** 1 College of Electronic Science and Technology and Key Laboratory of Optoelectronic Devices and Systems of Ministry of Education and Guangdong Province, Shenzhen University, Shenzhen 518060, China; 2 State Key Laboratory of Optoelectronic Materials and Technologies and School of Physics and Engineering, Sun Yat-Sen University, Guangzhou 510275, China; 3 Department of Physics and the Institute for Advanced Study, The Hong Kong University of Science and Technology, Hong Kong, China

## Abstract

Controlling of the lineshape of Fano resonance attracts much attention recently due to its wide capabilities for lasing, biosensing, slow-light applications and so on. However, the controllable Fano resonance always requires stringent alignment of complex symmetry-breaking structures and thus the manipulation could only be performed with limited degrees of freedom and narrow tuning range. Furthermore, there is no report so far on independent controlling of both the bright and dark modes in a single structure. Here, we semi-analytically show that the spectral position and linewidth of both the bright and dark modes can be tuned independently and/or simultaneously in a simple and symmetric metal-slit superlattice, and thus allowing for a free and continuous controlling of the lineshape of both the single and multiple Fano resonances. The independent controlling scheme is applicable for an extremely large electromagnetic spectrum range from optical to microwave frequencies, which is demonstrated by the numerical simulations with real metal and a microwave experiment. Our findings may provide convenient and flexible strategies for future tunable electromagnetic devices.

Optical Fano resonance has been a focus in nanophotonics field owing to its ultra-narrow linewidth which is available for switching^1^,^2^, lasing^3^,^4^,^5^,^6^, biosensing^7^,^8^,^9^,^10^, nonlinear optics, slow light application^11^,^12^,^13^ and so on. Fano resonance with an unusual asymmetric lineshape arises from the interference between a subradiant dark mode and a superradiant bright mode^14^,^15^. When the spectral position of the dark mode coincides with that of the bright mode, there will be an ultra-narrow transmission peak over a broad dip, which is considered as the classic analog of electromagnetically induced transparency (EIT)^16^,^17^,^18^,^19^. The system that supports Fano or EIT-like response usually requires complex unit cell geometry with symmetry-breaking structures such as the dipole quadrupole coupled metamaterials or metasurfaces^17^,^18^,^20^,^21^,^22^, asymmetric split ring resonators^23^,^24^,^25^,^26^, detuned resonator-pair^27^,^28^,^29^, waveguide-plasmon coupled systems^30^,^31^,^32^ and so on. Those structures rely on the near-field coupling and they require stringent alignment precision between nanostructure elements.

Manipulation of the lineshape of Fano resonance leads to a wide variety of intriguing physical transition phenomena^33^,^34^,^35^,^36^ and useful applications with large flexibility^9^,^11^,^37^,^38^. The controlling methods may include the variation of geometric parameters such as dislocated metal-bar pair^39^, multilayered metallic nanoshell^40^ and plasmonic/dielectric nanoclusters^41^,^42^,^43^. There are also schemes using active tunable materials such as phase-change material^44^ and graphene^45^,^46^,^47^. However, most of the previous studies, if not all, could control only one kind of the mode (bright mode or dark mode), while could not, to the best of our knowledge, independently control both the bright and dark modes on a single structure. Moreover, the manipulation of resonance lineshape in previous studies relies on the precise controlling of small separation between complex substructures in each unit cell, which leads to limited degrees of freedom and narrow tuning range.

On the other hand, metallic subwavelength arrays are widely studied in the past decade owing to their novel optical phenomena such as negative refraction^48^, super-resolution imaging^49^, and extraordinary optical transmission (EOT)^50^,^51^. The EOT peak for metallic subwavelength slit array can be attributed to the excitation of surface plasmon polariton (SPP) or the Fabry-Perot-like cavity modes inside the slits. Addition of slit number in one period forms the superlattice, which exhibits sharp dips over the broad transmission peak owing to the so called phase resonances^52^. In finite multiple-slit structure with the same unit cell as the superlattice, there exists a Fano resonance owing to the interference between the superradiant in-phase mode and subradiant out-of-phase mode as reported recently^53^,^54^. However, the general variation regularity of the spectral position as well as the linewidth of either the dark or bright mode is still unclear due to the lack of systematic study on the coupling mechanism between the slit cavity modes.

In this paper, we employ the subwavelength metal-slit superlattice to manipulate the Fano resonance with more degrees of freedom but in a much simpler way. We for the first time found that the far-field and near-field coupling mechanisms independently lead to the shifts in the spectral position of the in-phase mode and out-of-phase mode respectively, and thus the spectral positions of the bright and dark modes of Fano resonance can be separately controlled in a large range by the global and local slit distances respectively. In this way, the asymmetric factor of Fano resonances can be smoothly tuned from negative to positive value. The linewidth of both the bright and dark modes can also be tailored easily by the combination of slit width and superlattice’s period. Besides the full controlling of single Fano resonance, we can control the lineshape of multiple Fano resonances in a similar way. The proposed schemes are applicable in a wide frequency range from microwave to optical regime. Our findings may facilitate effective plasmon lasing applications by freely matching the sharp and broad resonances to the emission and absorption frequencies, respectively, of an arbitrary gain medium. It may be also useful for biosensors by matching resonance frequency to a particular molecule’s vibrational fingerprint^9^.

## Results

### Theoretical model for manipulation of Fano resonance

**Figure 1:**
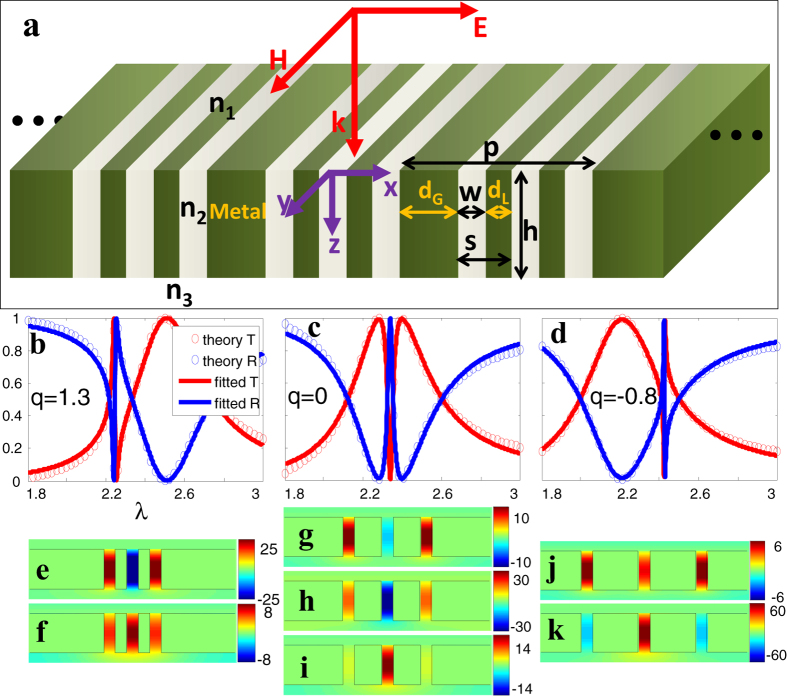
Schematic of the metal-slit superlattice structure; the Fano lineshapes and the field patterns at resonances. (**a**) Schematic of the metal-slit superlattice with slit number *m* = 3 in each supercell, global period *p*, local period *s*, slit width *w* and film thickness *h*. The local slit distance (between neighboring slits in one supercell) and global slit distance (between two supercells) are denoted as *d*_*L*_, *d*_*G*_, respectively. The refractive indexes above the metal film, in the slits area and below the metal film are n_1_, n_2_, n_3_, respectively. Plane wave normally illuminates the structure with transverse magnetic (TM) polarization. (**b–d**) the transmission (red) and reflection (blue) spectra of the structure as shown in (**a**) with parameters *p* = 1.8, *w* = 0.1 and (**b**) s = 0.2, (**c**) *s* = 0.336, and (**d**) *s* = 0.5, respectively. Wavelength and all the geometric parameters are normalized to film thickness *h*. The Fano lineshape exhibits different asymmetry factors: (**b**) q = 1.3, (**c**) q = 0, and (**d**) q = −1.8. Circle curves are the results of the theoretical model whereas the solid curves represent the Fano fitting results. The corresponding field patterns (H_y_) are illustrated in (**e–g**). The upper and lower panel of (**e/g**) show the field patterns at the left and right transmission peaks of (**b/d**) respectively. The upper, middle and lower panel of (**f**) show the field pattern at the left dip, middle peak and right dip for the reflection spectrum, respectively. All the field profiles is normalized to incident field amplitude.

Figure 1a illustrates the schematic of the metal-slit superlattice under study. A metallic film with thickness *h* is decorated with deep subwavelength slits (w≪λ) arranged as superlattice. The global and local period of the superlattice is *p* and *s*, respectively. The distance between neighboring supercells *d*_*G*_ and the distance between neighboring slits in one supercell *d*_*L*_ are the most important parameters for the manipulation of the Fano lineshape, and will be discussed in detail below. We analyze the electromagnetic (EM) response of the metallic superlattice with the help of a modal expansion method based on mode-matching theory^50^. In this method, the cavity mode in slit area is matched to the propagating plane waves outside the superlattice (see Supplementary Note 1). By imposing the boundary condition, we can semi-analytically write the zeroth order reflection and transmission coefficients of the superlattice embedded in air (*n*_1_ = *n*_2_ = *n*_3_ = 1) as,


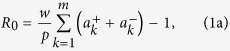



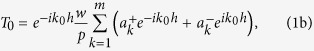


where, 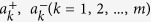 are the forward and backward amplitude coefficients of the cavity mode in the *k*^th^ slit of one supercell, which can be obtained by matrix inversion (see Equation (9) in Supplementary Notes 1).

It is well known that there are broad resonant transmission peaks and reflection dips for periodic subwavelength metal-slit lattices when the metal film is thick enough to support the Fabry-Perot (FP) resonances^50^. If we consider the superlattice arrangement of metallic slits with three slits in one supercell and different slit distances (*d*_*G*_≠*d*_*L*_), there will be an out-of-phase mode (upper panel of Fig. 1e) and an in-phase mode (lower panel of Fig. 1e) excited due to the mutual coupling between the cavity mode in each slit. The interference of the subradiant out-of-phase mode and the superradiant in-phase mode in each supercell leads to a Fano resonance with asymmetric lineshape (Fig. 1b,d) for both transmission and reflection spectra. The coupling between neighboring supercells allows an easy way to tune the Fano resonance with large degrees of freedom. From Fig. 1b–d, it is evident that, by simply varying the local period *s*, the asymmetric Fano lineshape can be tuned in different places relative to the broad resonance peak. When the sharp resonance is coincident with the broad resonance, the spectrum exhibits an EIT-like lineshape for the reflection spectrum (Fig. 1c). The field profile in the sharp reflection peak exhibits out-of-phase pattern with strong field enhancement (30-fold). These field profiles for the EIT-like lineshape are the superpositions of the eigenmodes with the forms of Equation (12) in Supplementary Note 1, as the dark mode overlaps with the bright mode. Although here the EIT-like peak exists for the reflection spectrum, we could obtain the similar EIT-like peak in transmission spectrum as the usual case in similar Babinet complementary structures.

The transmission spectral lineshape can be fitted by a Fano resonance modulated by Lorentz resonance using^54^,


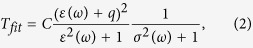


where, *C* is a normalization factor, *q* is the asymmetry parameter, 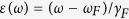, 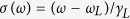, and 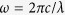 is the angular frequency, 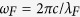 (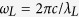), 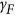 (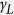) are the resonance frequency and spectral linewidth of the Fano (Lorentz) resonance, respectively. The fitted curves by Equation 2 (solid lines in Fig. 1b–d) are in good agreement with the semi-analytic results (circles in Fig. 1b–d). It is noted that the fitted asymmetry parameter *q* changes with the relative position between the bright and dark modes. When the dark mode is on the left (right) side of the bright mode (Fig. 1b,d), *q* is a finite positive (negative) number whose absolute value indicates the asymmetry degree of the Fano lineshape. The asymmetry factor equals exactly 0 when the dark mode coincides with the bright mode (Fig. 1c), exhibiting a symmetric EIT-like lineshape with an ultra-sharp peak over a broad dip in the reflection spectrum. In the later sections, we will use the analytical model to study the controlling of spectral position and linewidth of both the bright and dark modes by sweeping different geometric parameters.

### Manipulation of spectral position of the bright and dark modes

Let us first vary two types of slit distance (*d*_*G*_ and *d*_*L*_), which influence the coupling strength between the slit cavity modes and thus the spectral positions of the bright and dark modes. To clarify the origin of the sharp resonance and broad resonance, we ignore the cross coupling term between the bright in-phase mode and dark out-of-phase mode in the model expansion method, and calculate again the transmission spectra contributed independently by the bright and dark mode as shown in Supplementary Fig. S2. We observe that, there are a broad and a narrow transmission peak contributed independently by the bright and dark mode respectively.

**Figure 2:**
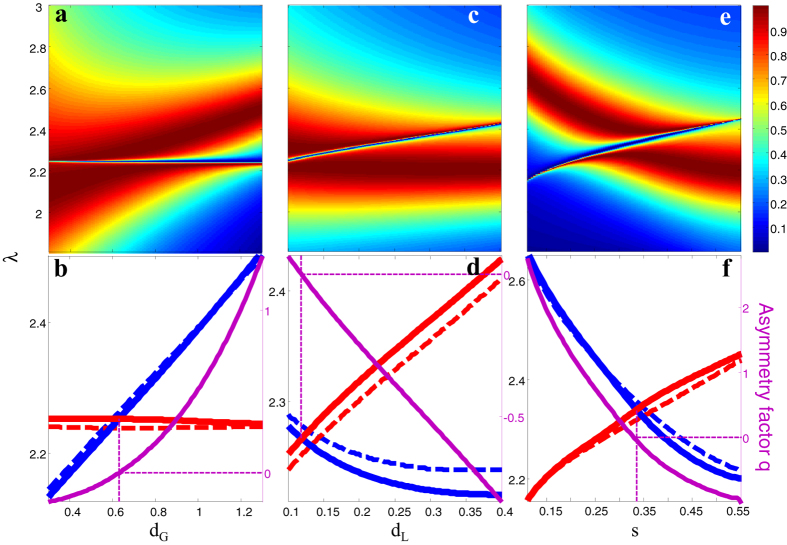
The evolution of Fano spectral profile with respect to different geometric parameters. In (**a,b**), *d*_*L*_ = 0.1 is fixed while *d*_*G*_ varying from 0.3 to 1.3; in (**c,d**), *d*_*G*_ = 0.7 is fixed while *d*_*L*_ varying from 0.1 to 0.4; in (**e,f**), p = 1.8 is fixed while *s* varying from 0.1 to 0.55. The slit width w = 0.1 for all cases. (**a**,**c**,**e**) show the phase-maps of transmittance |*T*_0_|^2^ as a function of wavelength *λ* and geometric parameter (**a**) *d*_*G*_, (**c**) *d*_*L*_ and (**e**) *s* respectively. The dashed blue and red curves in (**b**,**d**,**f**) are the resonance wavelengths of the bright and dark modes (the peak wavelengths in Supplementary Fig. S2) respectively; the solid blue and red curves are the Fano fitted parameters *λ*_L_, *λ*_F_, respectively. The solid purple curves are the Fano asymmetry factor *q* fitted by Equation (2) and the dashed purple curves indicate that the zero position of *q* represents the position where the dark mode coincides with the bright mode.

In Fig. 2a,b, the global distance between adjacent supercells *d*_*G*_ is varied from 0.3 to 1.3 (thereafter, all geometric parameters and wavelength are normalized to film thickness *h*) while the local distance *d*_*L*_ and slit width *w* are fixed. The in-phase bright mode shifts to longer wavelength linearly with the increasing of *d*_*G*_, while the position of the out-of-phase dark mode is remained at *λ*_0_ = 2.25 as indicated by Supplementary Fig. S2a,b. It can be explained by the far-field radiative dipolar coupling mechanism with a linear distance dependence^55^,^56^. The in-phase cavity modes in metal slits can be considered as transverse dipole array modes. The increased restoring force makes the blue-shift of the spectral position of bright mode with increasing of coupling strength (decreasing of global slit distance). The out-of-phase dark modes are localized modes with little radiation, therefore the far-field coupling is weak and the spectral position of dark mode is independent of the global distance. The actual position of *λ*_L_ (*λ*_F_) fitted by the Fano formula (solid curves in Fig. 2b,d,f)) has a little shift with respect to the position of the bright mode *λ*_b_ (dark mode *λ*_d_) (dashed curves in Fig. 2b,d,f)) due to the mutual interaction between the bright and dark modes. The fitted asymmetry factor *q* is also shown as a function of *d*_*G*_ (purple curves in Fig. 2b), where *q* continuously changes from negative to zero and finally a positive value when the bright mode changes from the shorter wavelength side of the dark mode to the longer wavelength side. When the bright mode coincides with the dark mode, the asymmetric factor equals to zero exactly and the reflection spectrum exhibits the EIT-like lineshape.

Next, let’s keep slit width *w* and global distance *d*_*G*_ unchanged, and inspect the transmission spectra for varying *d*_*L*_. From the Supplementary Fig. S2c,d, we see that, the spectral position of the bright mode is nearly unchanged with only a little spectral width shrink, but the resonance wavelength of dark mode increases linearly with the local slit distance *d*_*L*_. As a result, in the Fano lineshape (Fig. 2c) resulting from the interference between the bright and dark modes, the sharp resonance shifts to longer wavelength linearly with the local distance *d*_*L*_, while the broad resonance peak keeps its spectral position unchanged. Here, the local slit distance determine the near-field coupling strength between adjacent slit cavity modes. The dark mode is strongly influenced by near-field coupling due to the out-of-phase field pattern, while the bright mode is nearly unaffected by the near-field coupling. We note that, the asymmetric factor *q* linearly depends on *d*_*L*_ (solid purple line in Fig. 2d), which provides us a convenient way to modulate the asymmetry of Fano resonance profile.

In the above, we demonstrated the independent controlling of bright mode and dark mode by *d*_*G*_ and *d*_*L*_, respectively. Now, we discuss the simultaneous controlling of the bright mode and dark mode. We fix the slit width *w* and the global period *p*, and vary the local period *s* from 0.1 to 0.55 as shown in Fig. 2e,f. This is equivalent to decreasing global slit distance (*d*_*G*_ = *p*-*w*-2*s*) from 0 to 0.45 while increasing local slit distance (*d*_*L*_ = *s*-*w*) from 1.5 to 0.55 at the same time. Since the spectral position of bright (dark) mode is linearly dependent on *d*_*G*_ (*d*_*L*_) independently as we just discussed in the above, the simultaneous change of both *d*_*G*_ and *d*_*L*_ will lead to the simultaneous shift of the bright mode and dark mode (Fig. 2e,f) in opposite directions. As a result, we can simultaneously tune the dark and bright modes and obtain nearly arbitrary relative positions between the dark and bright modes by controlling solely the local period *s*.

Since we have successfully demonstrated the independent and simultaneous controlling of the bright mode and dark mode for single Fano resonance with ‘*m* = 3’ superlattice, we also attempt the challenging problem of controlling multiple Fano resonances. For this purpose, we consider a superlattice with more slits (m > 3) in one super cell (see Supplementary Notes 2 and Supplementary Figs S3 and S4). The semi-analytical results show that, the positions of multiple dark modes will simultaneously shift to longer wavelength with the increase of the local slit distance *d*_*L*_, and the shift speed of the wider asymmetric resonance is larger than the narrower one. Meanwhile, the single bright mode depends solely on the global distance *d*_*G*_. The dependence of the spectral position of either the bright or dark mode on geometric parameter is monotonous, which facilitates the implementation for practical applications.

### Manipulation of spectral linewidth of the bright and dark modes

**Figure 3:**
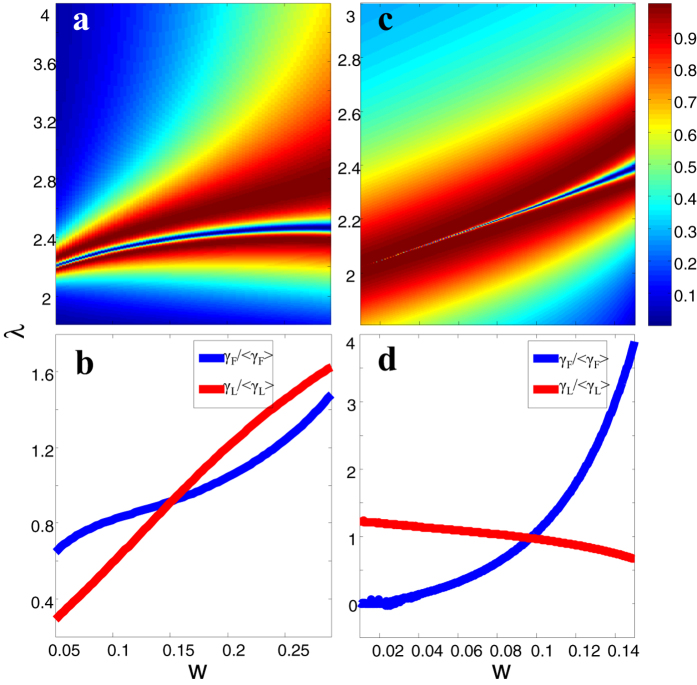
Fano profile variation with respect to slit width *w*. (**a,c**) transmittance spectra and (**b,d**) Fano fitted spectra linewidth *γ*_*F*_ and *γ*_*L*_ normalized to their average values. In (**a,b**), only slit width *w* is varied from 0.05 to 0.3 while p = 1.8, s = 0.3 are fixed. In (**c,d**), slit width *w* is varied from 0.01 to 0.15 while keeping s/w = 2, p/w = 12.

Now, let’s turn to study the influence of slit width *w* on the spectral profile of Fano resonance and discuss the tuning of spectral linewidth of both the bright and dark modes. For a single or periodic metallic subwavelength slit(s), the resonance linewidth increases with slit width due to increased radiation loss perpendicular to the metallic film^50^,^57^. For the case of slit-superlattice in our study, the slit width will influence both the dark and bright modes simultaneously as shown in Fig. 3a,b. Here, we choose appropriate *s* and *p* so that the dark mode and bright mode almost overlap and we can clearly see the linewidth of both the bright and dark modes. From the transmission spectra in Fig. 3a and the fitted Fano resonance linewidths *γ*_*F*_ and *γ*_*L*_ in Fig. 3b, we see that the spectral linewidths of both the bright (broad red peak) and dark modes (narrow blue dip) are significantly broadened when the slit width is increased. At the same time, the whole profile of Fano resonance shifts to longer wavelength, which is consistent with the previous works dealing with single and periodic slit systems^50^,^57^. However, in the slit-superlattice system, we have found more interesting phenomena that don’t exist in the common single or periodic slit system. In Fig. 3c,d, we vary slit width *w* and keep *w/p* and *s/p* unchanged. It is found that the narrow resonance dip is broadened as the slit width is increased, but the broad resonance peak sustains its spectral linewidth almost unchanged. It indicates that the duty-cycle *w/p* determines the spectral linewidth of the bright mode, whereas the slit width *w* solely determine the linewidth of dark mode. Thus it allows independent tuning of the linewidth of dark mode by varying the grating duty-cycle. Similarly it is also possible to independently tune the linewidth of bright mode by only changing parameter *p* as shown in Fig. 2a,c. Note that we can also control both the spectral linewidth and position of the Fano resonance in more degrees of freedom by parameter combinations as shown in Supplementary Fig. S5.

### Influence of dispersion and loss of real metal on the full controlling of Fano resonance

**Figure 4:**
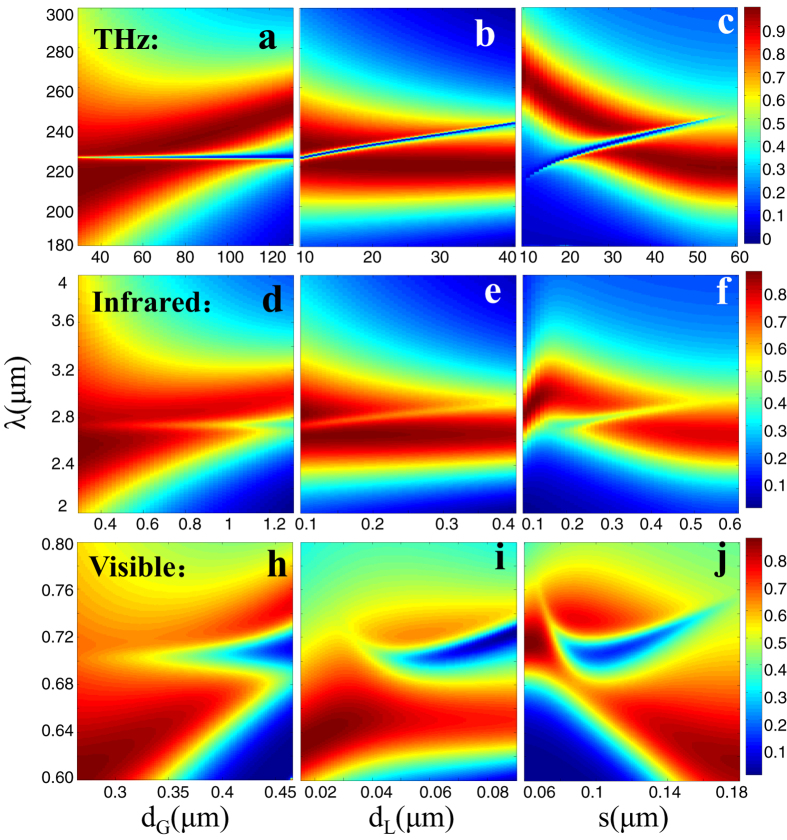
The evolution of Fano spectral profile with respect to different geometric parameters considering the loss and dispersion of metal at different electromagnetic spectral range calculated by FEM simulation. (**a–c**) are for Terahertz frequency range with Aluminum film thickness *h* = 100 μm, conductivity *σ* = 3.72e7 S/m. (**d–f**) are for infrared frequency range with silver film thickness *h* = 1 μm. (**h–j**) are for visible frequency range with silver film thickness h = 170 nm, w = 45 nm. The complex refractive index of silver in visible and infrared range is taken from the sampled experimental data^59^.

So far, the variation regularity of Fano resonance in metal-slit superlattice has been semi-analytically predicted by the ideal PEC model, in this part, we will validate the applicability of our proposed structure for real metals by the finite element method (FEM) simulation. In the microwave frequency range, the metal can be well described by PEC, thus the semi-analytical prediction is fully applicable for microwave range. In the terahertz (THz) frequency range, we consider the aluminum with finite conductivity *σ* = 3.72e7 S/m^58^, and numerically calculated the transmission spectra for varying *d*_*G*_, *d*_*L*_ and s in Fig. 4a–c respectively. We see that the spectral shape and position are similar with the theoretical results (Fig. 3a,c,e), although there are a little discrepancy (Supplementary Fig. S6b) for the resonance frequency and sharpness due to the influence of finite conductivity^57^.

When we move the frequency to optical frequency range (near-infrared and visible range), we consider the metal as silver whose complex refractive indexes are taken from Palik’s experimental data^59^. In the infrared range (Fig. 4d–f), the thickness of silver is scaled to 1 μm, and the slit width is *w* = 0.1*h* = 100nm. Those geometry parameters are large enough to be fabricated by nowadays nanotechnologies. From Fig. 4d–f, we see that the position of the bright (dark) mode is still separately dependent on *d*_*G*_ (*d*_*L*_). However, the overall resonance position shifts to longer wavelength. It is because, at infrared range, the gap surface plasmon modes with larger propagating constant (shorter effective wavelength) replace the cavity modes between PEC walls. It requires longer freespace wavelength to match the effective wavelength of the gap surface plasmon modes.

If we further reduce the thickness of silver film to *h* = 170nm, the operating frequency can be scaled to visible range. Here, the slit width *w* = 45nm, which is still within the fabrication limit. The transmission spectra in Fig. 4h–j shows that the sharp resonances broaden and the transmission minima become very shallow compared with the semi-analytical results in Fig. 2a,c,e. It is due to significantly enhanced intrinsic losses of metal in visible frequencies. The variation regularity of Fano profile with respect to *d*_*G*_, *d*_*L*_ and *s* still survive (Fig. 4h–j). Nevertheless, the tuning range is largely decreased due to due to the strong dispersion of silver in visible frequencies.

### Experimental verification of the controlling of Fano resonance in superlattice

**Figure 5:**
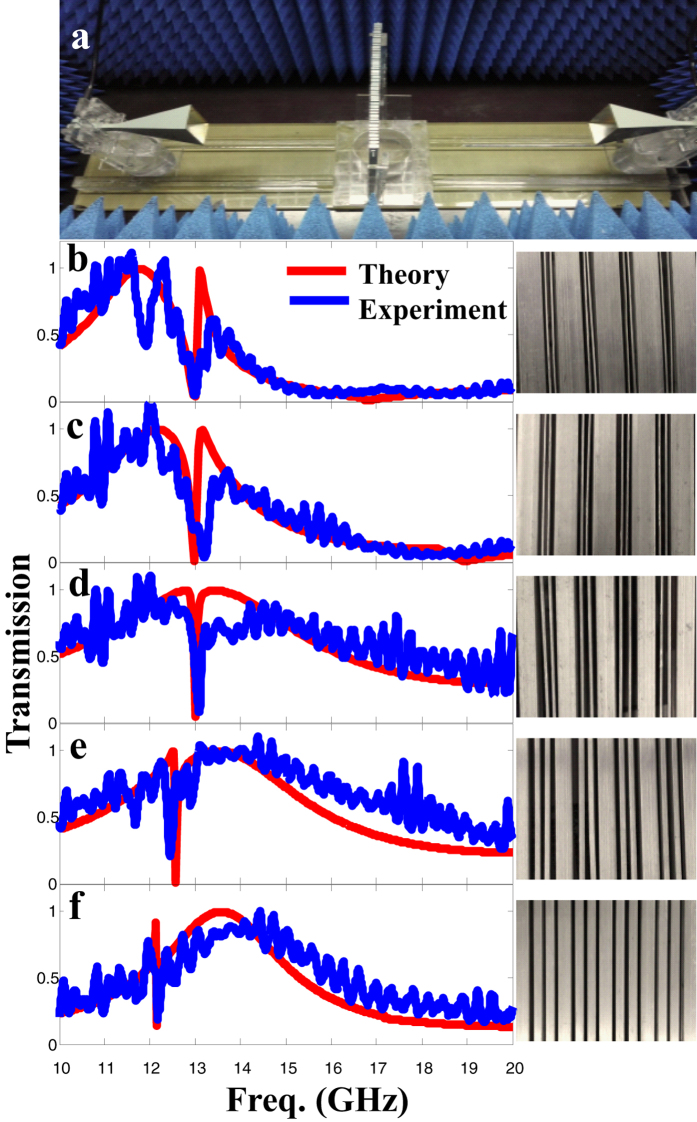
Experimental verification of the Full controlling of Fano resonance. (**a**) The photograph of the experimental setup. Two horn antennas connected to the vector network analyzer and the sample are placed inside a microwave anechoic chamber. Left panels of (**b–f**) show the experimental (blue) and theoretical (red) transmissions of metal-slit superlattices constructed by two types of Aluminum plates with different width *d*_*G*_ and *d*_*L*_ respectively. Right panels of (**b–f**) shows the corresponding photographs of the Aluminum superlattice samples with different *d*_*G*_ and *d*_*L*_ used in the experiment. The thickness and slit width of all the Aluminum superlattices is *h* = 10 mm, *w* = 1.3 mm respectively, while global and local distances are (**b**) *d*_*G*_ = 12 mm, *d*_*L*_ = 1 mm, (**c**) *d*_*G*_ = 10 mm, *d*_*L*_ = 1 mm, (**d**) *d*_*G*_ = 6 mm, *d*_*L*_ = 1 mm, (**e**) *d*_*G*_ = 6 mm, *d*_*L*_ = 2 mm, (**f**) *d*_*G*_ = 6 mm, *d*_*L*_ = 4 mm, respectively.

To further verify our theory, we make a simple experimental scheme in microwave frequencies to measure the transmission spectra of metal-slit superlattices with different slit distances (*d*_*G*_ and *d*_*L*_) as shown in Fig. 5. We measured the transmission spectra of five different samples under plane wave excitations in a Microwave anechoic chamber by the vector network analyzer (Fig. 5a). We carefully identify the broad peak representing the bright mode through the overall contour of the measured transmission spectra and the sharp dip representing the dark mode. We note that, there are many fluctuations in the experimental spectra of Fig. 5b–f due to the unavoidable Fabry-Perot (FP) resonances between the sample and antennas. We move the sample in different places and observe that, while a lot of shallow dips shift slightly, there is always a sharp dip sustaining its position unchanged. Then, we can identify the static sharp dip as the spectral position of the dark mode. We see that both the overall contour and the position of the sharp dip of the experimental spectra are consistent with the theoretical curves. In Fig. 5b–d, the three samples are of the same local distance *d*_*L*_ (width of the narrower Aluminum stripe), but different global distance *d*_G_ (width of the wider Aluminum stripe). The measured transmission spectra exhibit sharp dips at the same spectral position near 13 GHz, while the broad peak contour shifts to higher frequency (shorter wavelength) when global distance is decreasing. It indicates that the dark mode can be indeed independently controlled by global slit distance with fixed local slit distance. In Fig. 5d–f, the wider Aluminum stripes are of the same width (*d*_*G*_), while the width of the narrower stripes (*d*_*L*_) is increasing. As expected, the broad transmission peak contour share the same position near 13.5 GHz, while the sharp transmission dip shifts to lower frequency (longer wavelength). It indicates that, the dark mode could also be separately controlled by *d*_*L*_ with fixed *d*_*G*_. Thereby, the experimental results further validate the independent controlling of the spectral positions of the bright and dark modes by the global slit distance and the local slit distance, respectively.

## Discussion

It is intriguing that we can independently manipulate the sharp resonance and broad resonance with two independent parameters. It provides us with a very easy and flexible way to continuously tune the bright (dark) mode position for a large range at a given dark (bright) mode. Moreover, the linear dependency of *λ*_L_ (*λ*_F_) on *d*_*G*_ (*d*_*L*_) allows a linear modulation and thus further simplifies the tuning procedure. It also allows the continuous tuning of the asymmetric factor of Fano resonance, help us to obtain the desired Fano lineshape of any kind.

The independent and continuous controlling of the sharp and broad resonances have a wide range of practical applications such as nanoscale lasing and biosensing. For example, for the plasmonic nanolaser application, it is desirable to tune the broad resonance to overlap with the absorption frequency of the gain medium in order to enhance the energy conversion efficiency. And it is also essential to make the sharp resonance overlap with the emission frequency of the gain medium for the purpose of high lasing power and low threshold. Our proposal fulfills the criteria to match both the resonances to an arbitrary gain medium and thus providing an easy and effective way to design plasmonic lasing devices. The full controlling of Fano resonance also facilitates the biosensing application by tuning the subradiant resonance to match to the molecule’s vibrational fingerprints^9^.

In summary, by systematically investigating the EM wave response of the subwavelength metal-slit superlattice through the semi-analytic theory, numerical simulation and microwave experiment, we found a convenient and flexible way to independently manipulate the bright and dark modes of Fano resonances. The spectral position of the bright (dark) mode linearly depends on the global (local) slit distance separately owing to the far-field (near-filed) coupling. The spectral linewidth of the bright mode is determined by the duty-cycle *w/p*, while the linewidth of the dark mode is solely determined by slit width *w*. The asymmetry of the Fano lineshape can be continuously tuned so that we can easily obtain arbitrary spectral lineshapes such as EIT-like lineshape or Fano lineshape with desired asymmetric factor. The controlling of multiple Fano resonances can also be realized in a similar way with more slits in one supercell. The proposed controlling scheme for Fano resonance is applicable for an extremely wide EM spectrum range from microwave regime to optical frequencies. Our proposal are not restricted in metal-slit structures, but can readily extend to other structures such as metal or dielectric stripe superlattices. Our findings may lead to convenient and flexible applications in switching, sensing, lasing, slow light areas and so on.

## Methods

### Theory and simulation

The transmission and reflection coefficients of the subwavelength metal-slit superlattice with arbitrary slit number in one supercell are obtained semi-analytically by the model expansion theory. In that model, the cavity mode in slit area is matched to the propagating plane waves outside the grating by the boundary condition which requires the continuity of parallel *E*-field and *H*-filed components in each interface between grating and free space. We can first obtain the amplitude coefficients of cavity mode in slit area simply by a matrix inversion, and then directly obtain the transmission and reflection coefficients in terms of the amplitude coefficients of cavity mode. The simulation results for real metal are obtained by the finite element method implemented by COMSOL Multiphysics. The Aluminum is represented by a conductive model with finite conductivity *σ* = 3.72e7 S/m^58^ in Terahertz range. The complex refractive index of silver in optical frequencies are taken from Palik’s experimental data^59^.

### Sample fabrication and experiment

Each slit-superlattice sample is fabricated by aligning two types of commercially available Aluminum stripes with standard sizes. After drilling two holes in each side of all the aluminum stripes, we align them together with the help of two long screws for each sample in such a way that the adjacent aluminum stripes are separated by two shims with total thickness 1.3 mm. The total size of each sample is 300 mm×260 mm×10 mm. We fabricated five different samples and put them in a microwave anechoic chamber to measure the scattering parameters (S-parameters) by a vector network analyzer (Agilent E8362B PNA, 10 MHz-20 GHz). Two standard gain Ku-band horn antennas are used as transmitter and receiver. The distance between the two antennas are kept as 600 mm in order to ensure the planewave approximation. The transmission spectra of all the samples are normalized against the background radiation in which scattering parameters are measured without samples between the two horn antennas.

## Additional Information

**How to cite this article**: Deng, Z.-L. *et al.* Full controlling of Fano resonances in metal-slit superlattice. *Sci. Rep.* **5**, 18461; doi: 10.1038/srep18461 (2015).

## Supplementary Material

Supplementary Information
