# Supplementary material for: Full controlling of Fano resonances in metal-slit superlattice
Source: Sci Rep. 2015 Dec 18;5:18461. doi: 10.1038/srep18461 (PMC4683451; doi:10.1038/srep18461)
Supplement: Supplementary Information [file srep18461-s1.pdf]

Supplementary Information of
"Full controlling of Fano resonances in metal-slit superlattice"

Zi-Lan Deng[1], Natesan Yogesh[1], Xiao-Dong Chen[2], Wen-Jie Chen[3], Jian-Wen Dong[2], Zhengbiao Ouyang[1] and Guo Ping Wang[1,*]

[1]College of Electronic Science and Technology and Key Laboratory of Optoelectronic Devices and Systems of Ministry of Education and Guangdong Province, Shenzhen University, Shenzhen 518060, China

[2]State Key Laboratory of Optoelectronic Materials and Technologies and School of Physics and Engineering, Sun Yat-Sen University, Guangzhou 510275, China.

[3]Department of Physics and the Institute for Advanced Study, The Hong Kong University of Science and Technology, Hong Kong, China.

*Corresponding author: gpwang@szu.edu.cn

Supplementary Figures

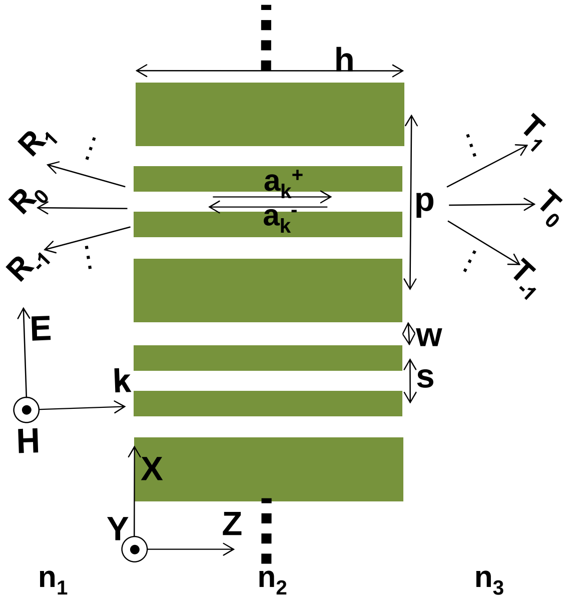

Supplementary Figure S1. Schematic of the structure of metal-slit superlattice analyzed by model expansion method. Transverse magnetic (TM) polarized plane wave incidents from left side with an arbitrary incident angle. There will be Fabry-Perot (FP)-like cavity mode (with amplitude coefficients a_k^+ and a_k^-) excited in each slit and transmitted and reflected plane wave with multiple diffraction orders ($R_{...-1, 0, 1...}$, $T_{...-1, 0, 1...}$) in free space.

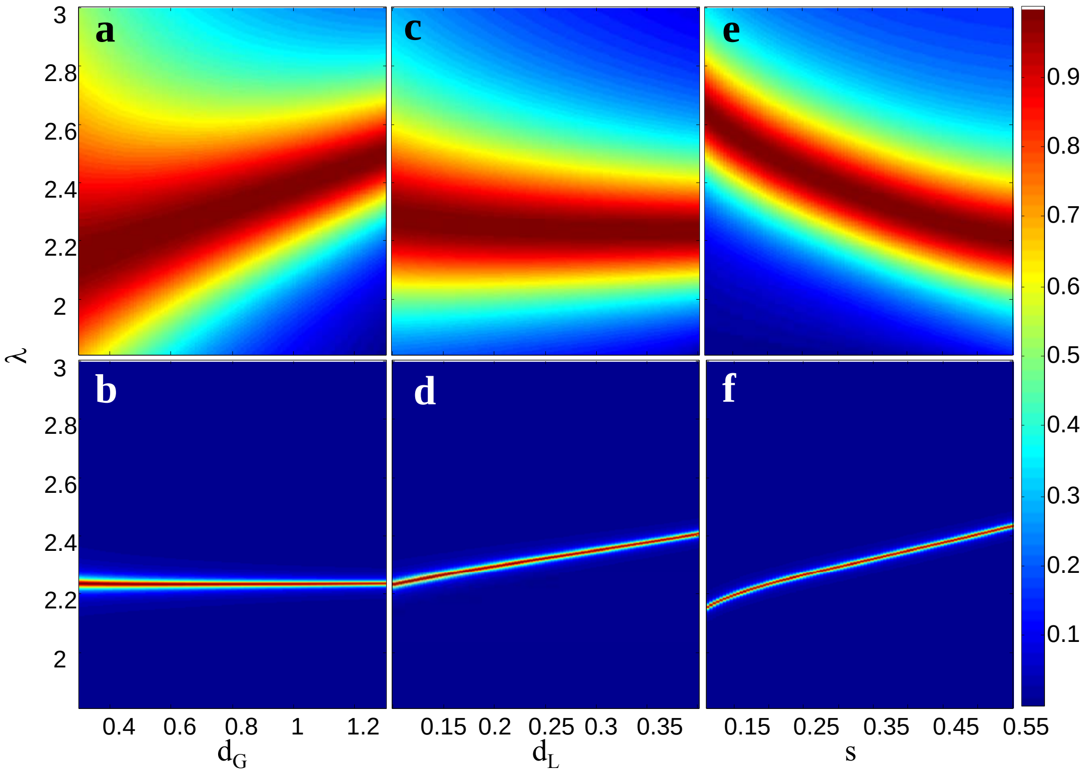

Supplementary Figure S2. The transmission spectra contributed separately by the in-phase bright mode (a, c, e) and the out-of-phase dark mode (b, d, f) calculated by the model expansion theory without considering the coupling terms between the bright and dark modes. Both the bright mode and the dark mode lead to transmission peaks but with significantly different spectral widths. In (a, b), w=0.1, d_L=0.1, and d_G is varied from 0.3 to 1.3. The broad peak in (a) shifts to longer wavelength while the position of the sharp peak in (b) stays unchanged. In (c, d), w=0.1, d_G=0.7, and d_L is varied from 0.1 to 0.4. The broad peak in (c) keeps the position unchanged while the sharp peak in (d) shifts to longer wavelength. In (e, f), w=0.1, p=1.8, and s is varied from 0.1 to 0.6. The broad peak in (e) shifts to shorter wavelength and the sharp peak in (f) shifts to longer wavelength simultaneously. The wavelength and all geometric parameters are normalized to film thickness h.

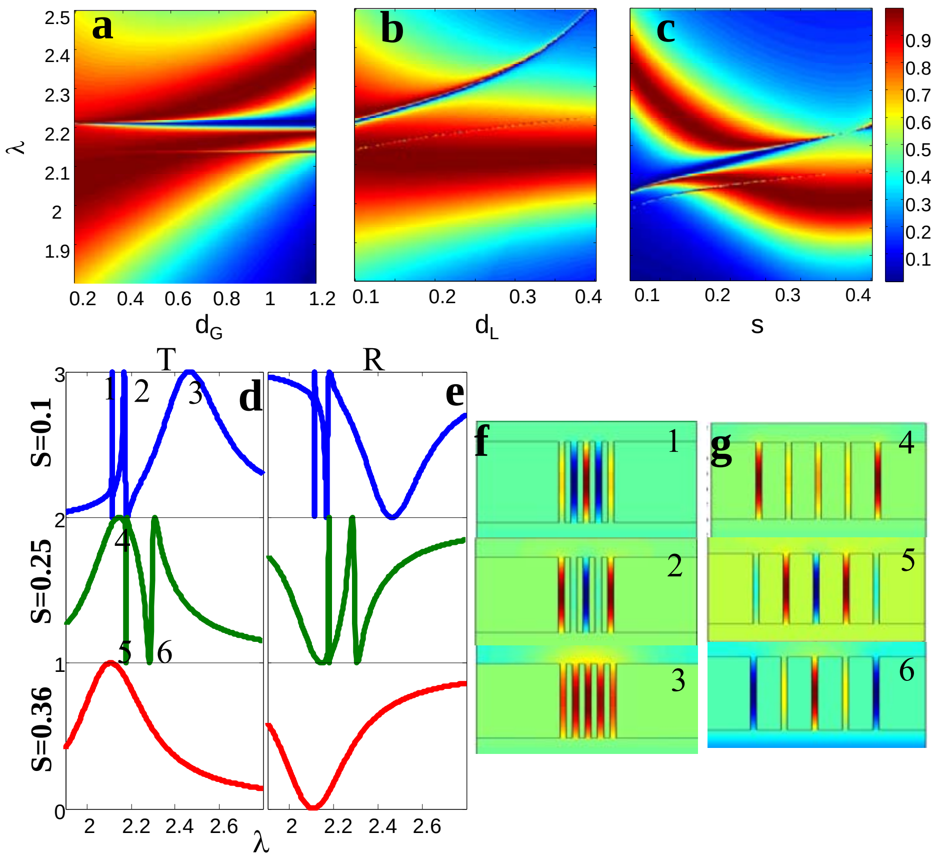

Supplementary Figure S3. The evolution of multiple Fano spectral profile in "*m*=5" superlattice with respect to different geometrical parameters (a) d_G, (b) d_L and (c) *s*. (d) and (e) show the transmittances and reflectance spectra (*p*=1.8, *w*=0.05) with different *s*=0.1, 0.25 and 0.36 respectively. Wavelength and all geometrical parameters are normalized to film thickness *h*. (f) shows the H_y field pattern at point 1, 2, 3 in the upper panel of (d); (g) shows the H_y field pattern at point 4, 5, 6 in the middle panel of (d). In the upper and middle panel of (d, e), there are 2 sharp resonances and 1 broad resonance. However, in the lowest panel of (d, e) there is only 1 broad resonances, since here *s*=*p*/*m* and the superlattice degrades into the ordinary periodic lattice.

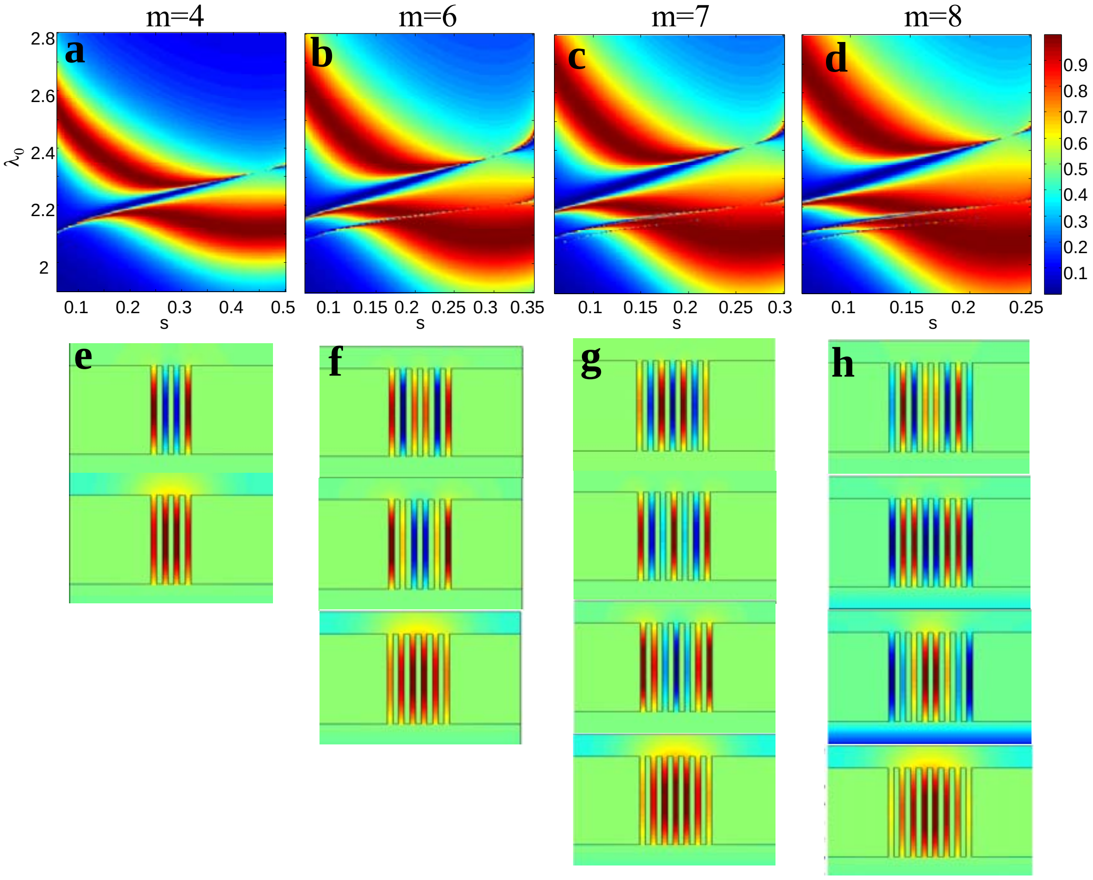

Supplementary Figure S4. The evolution of multiple Fano spectral lineshape with respect to local period *s* and the corresponding field pattern for different slit numbers (a, e) *m*=4, (b, f) *m*=6, (c, g) *m*=7, (d, h) *m*=8 in one supercell. Generally, there are one broad resonance and [(*m*-1)/2] sharp resonances for a superlattice with arbitrary *m*. Here, [.] is the largest integer operator. The field patterns of the broad resonances are in-phase as shown in the lowest panel in (e, f, g, and h). The field patterns of sharp resonances exhibit certain phase profiles and thus interfere destructively in the far field, leading to the sharp spectral linewidths.

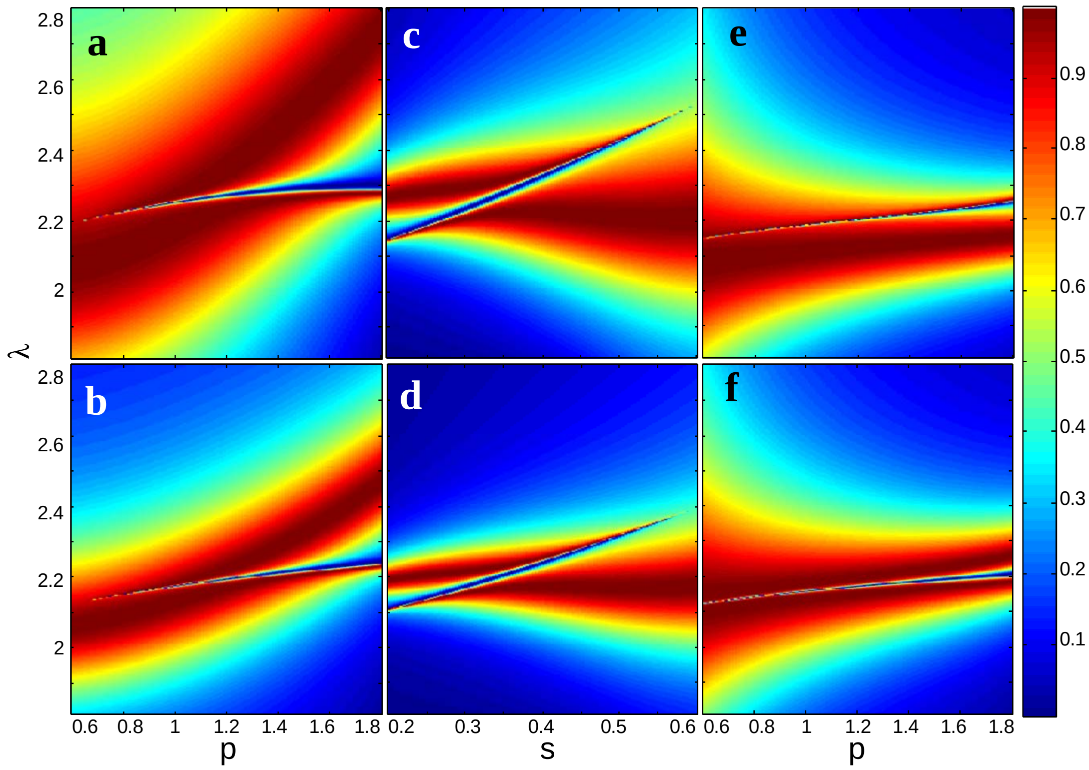

Supplementary Figure S5. **The Fano spectral profile variation with respect to different parameter combinations.** (a, b) are transmission spectra of the "*m*=3" superlattice with *s*=0.2 and *p* varying from 0.6 to 1.8 while keeping (a) *p/w*=10 and (b) *p/w*=20 respectively. (c, d) show the transmission spectra of the superlattice with *p*=1.8 and *s* varying from 0.2 to 0.6 while keeping (c) *s/w*=5 and (d) *s/w*=10 respectively. (e, f) illustrate the transmission spectra of the superlattice with w=0.05 and p varying from 0.6 to 1.8 while keeping (e) *p/s*=4 and (f) *p/s*=6 respectively.

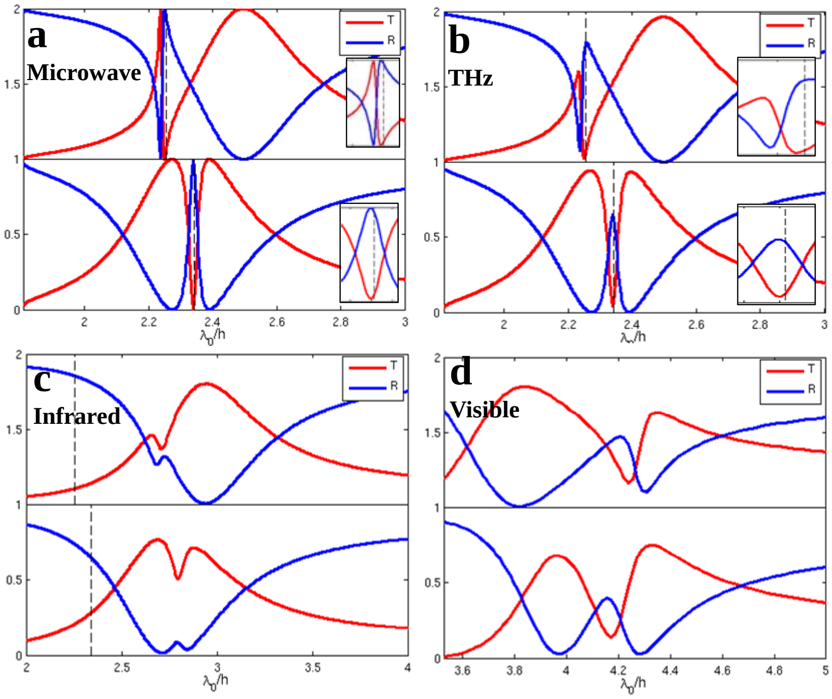

Supplementary Figure S6. The asymmetric Fano spectral lineshape and EIT-like lineshape for superlattice consisting of real metal with finite conductivity or finite permittivity in different electromagnetic spectral range. In (a, b) the metal is considered as Aluminum with conductivity σ=3.72e7 S/m. The thickness of metal is (a) h=10 mm and (b) h=100 μm respectively. In (c, d) the metal is considered as silver with Drude-Lorentz model. The thickness of metal film is (c) h=1 μm and (d) h=170 nm respectively. The dashed line indicates the position of the sharp Fano resonance predicted by model expansion theory which consider the metal as PEC. In microwave range, the spectral shape is identical to the results by the expansion theory with a negligible difference of the spectral position of the dark mode. In Terahertz range, the lineshape is still similar with the theoretic model. The finite conductivity makes a little shift of the dark mode and a reduction of the peak value of resonance. In infrared range, the overall spectral position shifts to longer wavelength and the resonance shapes become very shallow. In visible range, the dark modes are largely broadened due to strong loss of metal in optical frequencies.

Supplementary Notes

Supplementary Note 1. Model expansion theory of metal-slit superlattice

Let us consider the general case as shown in Fig. S1. There are generally m slits with width w and local period s in each supercell. The global period and thickness of metallic film is p and h respectively. The dielectric media of the superstrate, slit area

and substrate are of the refractive index $n_1=\sqrt{\varepsilon_1\mu_1}$, $n_2=\sqrt{\varepsilon_2\mu_2}$ and $n_3=\sqrt{\varepsilon_3\mu_3}$ respectively. A plane wave with arbitrary incident angle (or parallel wavevector k_{x0}) and TM polarization (Magnetic fields is always parallel to the slit direction) illuminates the structure from the superstrate. Owing to the diffraction effect of grating, the electromagnetic (EM) field in superstrate and substrate can be expanded as the plane waves of all diffraction orders. As a result, the transverse EM field components in superstrate and substrate have the forms as,

$$H_{1y}=H_0\left(\mathrm{e}^{ik_{x0}x+ik_{z0}^{(1)}z}-\sum_{n=-\infty}^{\infty}R_n\,\mathrm{e}^{ik_{xn}x-ik_{zn}^{(1)}z}\right),\qquad (1a)$$

$$E_{1x}=\frac{1}{i\omega\varepsilon_1\varepsilon_0}\frac{\partial H_y}{\partial z}=\frac{k_{z0}^{(1)}H_0}{\omega\varepsilon_1\varepsilon_0}\left(\mathrm{e}^{ik_{x0}x+ik_{z0}^{(1)}z}+\sum_{n=-\infty}^{\infty}R_n\frac{k_{zn}^{(1)}}{k_{z0}^{(1)}}\mathrm{e}^{ik_{xn}x-ik_{zn}^{(1)}z}\right),\qquad (1b)$$

$$H_{3y}=H_0\sum_{n=-\infty}^{\infty}T_n\,\mathrm{e}^{ik_{xn}x+ik_{zn}^{(3)}z},\qquad (2a)$$

$$E_{3x}=\frac{1}{i\omega\varepsilon_3\varepsilon_0}\frac{\partial H_y}{\partial z}=\frac{k_{z0}^{(3)}H_0}{\omega\varepsilon_3\varepsilon_0}\sum_{n=-\infty}^{\infty}T_n\frac{k_{zn}^{(3)}}{k_{z0}^{(3)}}\mathrm{e}^{ik_{xn}x+ik_{zn}^{(3)}z},\qquad (2b)$$

where, (k_{x0}, $k_{z0}^{(1)}=\sqrt{n_1^2k_0^2-k_{x0}^2}$) is the wavevector of incident plane wave, and ($k_{xn}=k_{x0}+2n\pi/p$, $k_{zn}^{(j)}=\sqrt{n_j^2k_0^2-k_{xn}^2}$) (j=1,3) is the wavevector of the n-th diffraction order in superstrate and substrate respectively. T_n and R_n are the complex transmission and reflection coefficients for the n-th diffraction order respectively. ε_0 is the permittivity of vacuum; ω is the angular frequency and H_0 is the amplitude of incident wave.

In the slit area, there are generally multiple waveguide modes in each metallic slit. However, for the deep subwavelength slit, the higher order modes are in cut-off range, and hence only the fundamental mode needs to be considered. For the m slits in each

supercell, the complex amplitude coefficients for each slit may be different due to their mutual coupling. We assume a_k^+ (k=1,2, …, m) and a_k^- are the forward and backward amplitude coefficient of the k-th slit in each supercell respectively, then the transverse EM field components in metallic slit area are,

$$H_y = H_0 \sum_{k=1}^{m} M_k\left(x\right)\left(a_k^+ e^{in_2k_0z} - a_k^- e^{-in_2k_0z}\right), \text{ for(-h<z<0)} \tag{3a}$$

$$E_x = \frac{1}{i\omega\varepsilon_2\varepsilon_0}\frac{\partial H_y}{\partial z} = \frac{n_2k_0H_0}{\omega\varepsilon_2\varepsilon_0}\sum_{k=1}^{m} M_k\left(x\right)\left(a_k^+ e^{in_2k_0z} + a_k^- e^{-in_2k_0z}\right), \text{ for(-h} \le \text{z} \le \text{0)} \tag{3b}$$

where, $M_k\left(x\right) = rect\left(\left(x - \left(k - \frac{m+1}{2}\right)s\right)/w\right)$ is the field form in the k-th slit, rect(.) is the rectangular function, and the amplitude coefficients a_k^+ and a_k^- are unknown variables which could be determined by the boundary condition requiring that the parallel EM field components should be continuous at all interfaces.

At the z=0 interface, the boundary condition yields,

$$\mathrm{e}^{ik_{x0}x} - \sum_{n=-\infty}^{\infty} R_n \,\mathrm{e}^{ik_{xn}x} = \sum_{k=1}^{m} M_k\left(x\right)\left(a_k^+ - a_k^-\right), \tag{4a}$$

$$\mathrm{e}^{ik_{x0}x} + \sum_{n=-\infty}^{\infty} R_n \frac{k_{zn}^{(1)}}{k_{z0}^{(1)}}\mathrm{e}^{ik_{xn}x} = \frac{\varepsilon_1}{\varepsilon_2}\frac{n_2k_0}{k_{z0}^{(1)}}\sum_{k=1}^{m} M_k\left(x\right)\left(a_k^+ + a_k^-\right), \tag{4b}$$

and at the $z=h$ interface, the boundary condition yields,

$$\sum_{n=-\infty}^{\infty} T_n \,\mathrm{e}^{ik_{xn}x + ik_{zn}^{(3)}h} = \sum_{k=1}^{m} M_k\left(x\right)\left(a_k^+ e^{in_2k_0h} - a_k^- e^{-in_2k_0h}\right), \tag{5a}$$

$$\sum_{n=-\infty}^{\infty} T_n \frac{k_{zn}^{(3)}}{k_{z0}^{(3)}}\mathrm{e}^{ik_{xn}x + ik_{zn}^{(3)}h} = \frac{\varepsilon_3}{\varepsilon_2}\frac{n_2k_0}{k_{z0}^{(3)}}\sum_{k=1}^{m} M_k\left(x\right)\left(a_k^+ e^{in_2k_0h} + a_k^- e^{-in_2k_0h}\right), \tag{5b}$$

Considering the orthogonality of different diffraction orders, we multiply Eq. (4b), (5b) by $\mathrm{e}^{-ik_{xn}x}$ and then integrate over $-p/2<x<p/2$ respectively. It yields,

$$p\delta_{n0}+\frac{k_{zn}^{(1)}}{k_{z0}^{(1)}}pR_n=\frac{\varepsilon_1}{\varepsilon_2}\frac{n_2k_0}{k_{z0}^{(1)}}w\sin c\left(\frac{k_{xn}w}{2}\right)\sum_{k=1}^{m}\mathrm{e}^{-i\left(k-\frac{m+1}{2}\right)k_{xn}s}\left(a_k^++a_k^-\right), \tag{6a}$$

$$\frac{k_{zn}^{(3)}}{k_{z0}^{(3)}}pT_ne^{ik_{zn}^{(3)}h}=\frac{\varepsilon_3}{\varepsilon_2}\frac{n_2k_0}{k_{z0}^{(3)}}w\sin c\left(\frac{k_{xn}w}{2}\right)\sum_{k=1}^{m}e^{-i\left(k-\frac{m+1}{2}\right)k_{xn}s}\left(a_k^+e^{in_2k_0h}+a_k^-e^{-in_2k_0h}\right), \tag{6b}$$

Thus, R_n, T_n can be expressed in terms of a_k^+, a_k^- as,

$$R_n=-\frac{k_{z0}^{(1)}}{k_{zn}^{(1)}}\delta_{n0}+\frac{\varepsilon_1}{\varepsilon_2}\frac{n_2k_0}{k_{zn}^{(1)}}\frac{w}{p}\sin c\left(\frac{k_{xn}w}{2}\right)\sum_{k=1}^{m}\mathrm{e}^{-i\left(k-\frac{m+1}{2}\right)k_{xn}s}\left(a_k^++a_k^-\right), \tag{7a}$$

$$T_n=e^{-ik_{zn}^{(3)}h}\frac{\varepsilon_3}{\varepsilon_2}\frac{n_2k_0}{k_{zn}^{(3)}}\frac{w}{p}\sin c\left(\frac{k_{xn}W}{2}\right)\sum_{k=1}^{m}\mathrm{e}^{-i\left(k-\frac{m+1}{2}\right)k_{xn}s}\left(a_k^+e^{-in_2k_0h}+a_k^-e^{in_2k_0h}\right), \tag{7b}$$

Multiplying Eq. (4a), (5a) by $M_{k'}(x)$ and then integrating in terms of x in region $-p/2<x<-p/2$ respectively, it yields,

$$e^{i\left(k'-\frac{m+1}{2}\right)k_{x0}s}\sin c\left(\frac{k_{x0}w}{2}\right)=\sum_{n=-\infty}^{\infty}R_n\,\mathrm{e}^{i\left(k'-\frac{m+1}{2}\right)k_{xn}s}\sin c\left(\frac{k_{xn}w}{2}\right)+a_{k'}^+-a_{k'}^-, \tag{8a}$$

$$0=\sum_{n=-\infty}^{\infty}T_n\,\mathrm{e}^{ik_{zn}^{(3)}h}\,\mathrm{e}^{i\left(k'-\frac{m+1}{2}\right)k_{xn}s}\sin c\left(\frac{k_{xn}w}{2}\right)-\left(a_1^+\,\mathrm{e}^{in_2k_0h}-a_1^-\,\mathrm{e}^{-in_2k_0h}\right), \tag{8b}$$

Substituting (7a, 7b) into (8a, 8b), respectively, it yields,

$$2e^{i\left(k'-\frac{m+1}{2}\right)k_{x0}s}\sin c\left(\frac{k_{x0}w}{2}\right)=\frac{\varepsilon_1}{\varepsilon_2}\frac{w}{p}\sum_{k=1}^{m}\left[\left(a_k^++a_k^-\right)\sum_{n=-\infty}^{\infty}\frac{n_2k_0}{k_{zn}^{(1)}}\mathrm{e}^{i(k'-k)k_{xn}s}\sin c^2\left(\frac{k_{xn}w}{2}\right)\right]+\left(a_{k'}^+-a_{k'}^-\right), \tag{9a}$$

$$0=\frac{\varepsilon_3}{\varepsilon_2}\frac{w}{p}\sum_{k=1}^{m}\left[\left(a_k^+\,\mathrm{e}^{in_2k_0h}+a_k^-\,\mathrm{e}^{-in_2k_0h}\right)\sum_{n=-\infty}^{\infty}\frac{n_2k_0}{k_{zn}^{(3)}}\mathrm{e}^{i(k'-k)k_{xn}s}\sin c^2\left(\frac{k_{xn}w}{2}\right)\right]-\left(a_{k'}^+\,\mathrm{e}^{in_2k_0h}-a_{k'}^-\,\mathrm{e}^{-in_2k_0h}\right), \tag{9b}$$

From Eq. (9), we can build a $2m\times2m$ matrix **M** with elements,

$$\mathbf{e}_{2k'-1,2k-1}=S_{k'-k}^r+\delta_{kk'},\quad\left(k'=1,\ 2,\ ...,\ m;\ k=1,\ 2,\ ...,\ m\right) \tag{10a}$$

$$\mathbf{e}_{2k'-1,2k}=S_{k'-k}^r-\delta_{kk'}, \tag{10b}$$

$$\mathbf{e}_{2k',2k-1}=\left(S_{k'-k}^t-\delta_{kk'}\right)e^{in_2k_0h}, \tag{10c}$$

$$\mathbf{e}_{2k',2k}=\left(S_{k'-k}^t+\delta_{kk'}\right)e^{-in_2k_0h}. \tag{10d}$$

where, $S_k^r=\frac{\varepsilon_1}{\varepsilon_2}\frac{w}{p}\sum_{n=-\infty}^{\infty}\frac{n_2k_0}{k_{zn}^{(1)}}\mathrm{e}^{ikk_{xn}s}\sin c^2\left(\frac{k_{xn}w}{2}\right)$, $S_k^t=\frac{\varepsilon_3}{\varepsilon_2}\frac{w}{p}\sum_{n=-\infty}^{\infty}\frac{n_2k_0}{k_{zn}^{(3)}}\mathrm{e}^{ikk_{xn}s}\sin c^2\left(\frac{k_{xn}w}{2}\right)$.

Then, the amplitude coefficients of cavity modes in metallic slits $\mathbf{a}=\left(a_1^+,a_1^-,a_2^+,a_2^-,...,a_m^+,a_m^-\right)^T$ can be solved by linear matrix equation, $\mathbf{a}=\mathbf{M}^{-1}\mathbf{b}$, where, $\mathbf{b}=\left(b_1,0,b_2 0,...,b_m,0\right)^T$ and $b_k=2e^{i\left(k-\frac{m+1}{2}\right)k_{x0}s}\sin c\left(\frac{k_{x0}w}{2}\right)$, $\left(k=1,\ 2,\ ...,\ m\right)$.

Finally, substituting the solved amplitude coefficients back into (7), we can readily obtain the reflection and transmission coefficients for arbitrary diffraction orders.

Let's consider the m=3 superlattice and the normal incidence (k_{x0}=0) case which are mainly discussed in the main text. Without loss of generality, we set both the superstrate and substrate as air (n_1=n_3=1). Owing to the symmetry of the configuration, $a_1^\pm\equiv a_3^\pm$ is always satisfied, thus we only need to solve a 4x4 matrix. The matrix **M** can be analytically written as,

$$\mathbf{M}_{m=3}=\begin{pmatrix} S_0+1 & S_0-1 & S_{-1} & S_{-1} \\ \left(S_0-1\right)e^{in_2k_0h} & \left(S_0+1\right)e^{-in_2k_0h} & S_{-1}e^{in_2k_0h} & S_{-1}e^{-in_2k_0h} \\ S_1 & S_1 & S_0+1 & S_0-1 \\ S_1e^{in_2k_0h} & S_1e^{-in_2k_0h} & \left(S_0+1\right)e^{in_2k_0h} & \left(S_0-1\right)e^{-in_2k_0h} \end{pmatrix} \quad (11)$$

where, $S_k=\sum_{n=-\infty}^{\infty}\frac{k_0\sin\left(n\pi w/p\right)\exp\left(i2kn\pi s/p\right)}{n\pi n_2\sqrt{k_0^2-\left(2n\pi/p\right)^2}}$, $(k=0,\pm1)$,

and, $\mathbf{b}=\left(2,\ 0,\ 2,\ 0\right)^T$.

Due to the symmetry of the structure and source, the normal incident planewave can excite two eigenmodes with even symmetry with in-phase and out-of-phase profile respectively. Their field distributions have the forms as [1],

$$E_1\left(x\right)=\frac{1}{2}rect\left(\frac{x-s}{w}\right)+\frac{1}{\sqrt{2}}rect\left(\frac{x}{w}\right)+\frac{1}{2}rect\left(\frac{x+s}{w}\right), \quad (12a)$$

$$E_3\left(x\right)=\frac{1}{2}rect\left(\frac{x-s}{w}\right)-\frac{1}{\sqrt{2}}rect\left(\frac{x}{w}\right)+\frac{1}{2}rect\left(\frac{x+s}{w}\right), \quad (12b)$$

We note that, in Ref. [1], the authors matches the coefficients ($c_1^{\pm}$, $c_3^{\pm}$) of the above modes with the propagating planewave to determine the transmission cross section in finite slits system. Their method needs to know the eigenmode profile of multiple-slit system before hand, which will be tedious especially when the slit number (in one supercell for our case) become very large. Instead, we choose an alternative mode matching technique by using amplitude coefficients ($a_1^{\pm}$, $a_2^{\pm}$) for each slit. In this case, it is unnecessary to know the eigenmode profile beforehand, and can be extended to structures with even more slits in one supercell in a straightforward way. By a little bit algebraic deduction, we could obtain the relationship between these two sets of coefficients as,

$$\begin{pmatrix} a_1^{\pm} \\ a_2^{\pm} \end{pmatrix} = \begin{pmatrix} 1/2 & 1/2 \\ 1/\sqrt{2} & -1/\sqrt{2} \end{pmatrix} \begin{pmatrix} c_1^{\pm} \\ c_3^{\pm} \end{pmatrix}, \tag{13}$$

As a result, we can still analyze the influence of different eigenmodes on the overall reflection and transmission by a simple matrix operation.

Supplementary Note 2. Multiple Fano resonances and multiple EIT-like spectra in superlattice with arbitrary number of slits in one supercell

Besides the superlattice with 3 slits in one supercell which exhibits easily controllable Fano resonance, superlattice with more slits (m>3) in one supercell also support Fano resonances with a variety of manipulation ways. Note that, the Fano resonance in “m=2” superlattice only exists under oblique incidence [2] since the out-of-phase mode with pattern “+-” has odd symmetry but the structure and normal incident source possess mirror symmetry. For “$m\geqslant 3$” superlattice, there exist out-of-phase

modes with even symmetry and can be excited by the normal incident plane wave. There will be generally [(m-1)/2] out-of-phase even modes for an m-superlattice, and thus [(m-1)/2] Fano resonances in a single structure.

Supplementary Figure S3 shows the case of "m=5" superlattice, where there are two sharp asymmetric resonance dips and one broad resonance peak. The positions of both the two sharp resonances stay unchanged while the broad resonance shits to longer wavelength when the global slit spacing d_G increases as shown in Supplementary Fig. S3a. The two sharp resonances shifts to longer wavelength together while the broad peak keeps its position when local slit spacing d_L increases as shown in Fig. S3 (b). Moreover, the shift speed of the wider asymmetric resonance is larger than the narrow one. When s is varied from 0.06 to 0.4 while other parameters are fixed, the two sharp resonances shift to short wavelength and at the same time the broad resonance shifts to longer wavelength. The multiple sharp resonances can be tuned to overlap with the broad resonance to form a multiple EIT-like lineshape, which is an interesting phenomenon studied widely in recent years.

Supplementary Figure S4 shows the transmission spectra and field patterns for multiple numbers of slit in one supercell and we have found that the positions of the multiple sharp resonances can be tuned to be at any places relative to the broad resonance by the local period s. And the dependence of the spectral position on parameter s is monotonous, which facilitates the implementation for practical applications.

Supplementary Note 3. Manipulation of Fano resonance by certain parameter combinations of the superlattice

Besides the simple tuning of a single parameter of the superlattice, the tuning of several parameters together can may provide more degrees of freedom and larger tuning range different ways of the manipulation of the Fano resonance as we have already seen in Fig. 3c in the maintext. In Supplementary Fig. S5, we simultaneously vary two of the three typical parameters *w*, *s*, *p*, but keeps its ratio unchanged to study the combined effect. From Supplementary Fig. S5a-5b, we see that it is possible to separately tune the position of bright mode while keeping the dark mode nearly unchanged by varying both *p* and w increase with fixed radio *p/w*. The spectral linewidth position of the bright mode keeps unchanged when both *p* and w increase, and the larger the *p*/*w*, the smaller the linewidth of the bright mode. It indicates that the spectral width of bright mode is indeed determined by the ratio *p*/*w*. When both *s* and *w* are increasing (s/w is fixed), the spectral position of bright mode (red peaks in Fig. 4c-4d)) is unchanged whereas the dark mode (blue dips in Supplementary Fig. S5c-5d)) shifts to longer wavelength. It is different from Fig. 2c that, here the dark mode can cross the whole lineshape of the bright mode, and thus allowing for an even larger tuning range for the dark mode. Moreover, the linewidth of bright mode broadens due to the increase of the overall duty-cycle. Comparing Supplementary Fig. 5c and 5d, we found that different ratio *s/w* makes different overall spectral linewidth, providing a convenient way to separately manipulate the spectral linewidth and relative spectral position. The relative position between dark mode and bright mode is

mainly determined by the ratio *p*/*s* as indicated in Supplementary Fig. S5e-5f. When *p*/*s*=6 (Supplementary Fig. S5f), the dark mode overlaps with the bright mode for any *p* (or *s*) values. Similarly, the dark mode will be above (Supplementary Fig. S5e) or below the dark mode when *p*/*s* is smaller or larger than 6. It provides us a clear guideline to obtain an EIT-like lineshape or a Fano lineshape with particular asymmetry factor.

References

1. S. Chen, S. Jin, and R. Gordon, "Subdiffraction Focusing Enabled by a Fano Resonance," Physical Review X **4**, 031021 (2014).
2. L. Verslegers, Z. Yu, Z. Ruan, P. B. Catrysse, and S. Fan, "From Electromagnetically Induced Transparency to Superscattering with a Single Structure: A Coupled-Mode Theory for Doubly Resonant Structures," Phys. Rev. Lett. **108**, 083902 (2012).
